# Digital healthcare as a solution for global challenges: A call for action

**DOI:** 10.1111/aogs.15066

**Published:** 2025-01-28

**Authors:** Mireille N. Bekker, Olof Stephansson, Nerea Maiz, Michèle van der Kemp, Kees Ahaus, Arie Franx

**Affiliations:** ^1^ Department of Obstetrics and Gynecology University Medical Centre Utrecht, Utrecht University Utrecht The Netherlands; ^2^ Department of Medicine Solna, Karolinska Institutet and Karolinska University Hospital Stockholm Sweden; ^3^ Obstetrics Department Hospital Universitari Val d'Hebron Barcelona Spain; ^4^ VDKMP Alliance Management, Value Based Healthcare Clinical Leadership & Care Redesign Amsterdam The Netherlands; ^5^ Health Services Management & Organization, Erasmus School of Health Policy and Management Erasmus Universiteit Rotterdam Rotterdam The Netherlands; ^6^ Department of Obstetrics and Gynecology Erasmus Medical Centre Rotterdam The Netherlands

In an era of rising technological advancements, healthcare systems worldwide are facing a coincidence of challenges: a growing shortage of healthcare professionals, unsustainable growth of the costs of care, and an alarming rise in chronic conditions. The global healthcare workforce is stretched to its limits, with the World Health Organization (WHO) projecting a shortfall of 10 million healthcare workers by 2030.^1^ Compounding this crisis are aging populations and the rising prevalence of chronic conditions, such as diabetes and cardiovascular diseases. The global economic burden of diabetes alone is expected to surpass $845 billion annually by 2045.^2^

These pressures demand innovative solutions. Digital health care—enabled by, for example, telemonitoring, artificial intelligence, and remote care platforms—emerges as a promising response. However, despite its potential to revolutionize care delivery, its implementation remains scarce. Research is urgently needed to validate its safety, efficacy, and cost‐effectiveness, ensuring sustainable integration into healthcare systems.

## 1 THE PROMISE OF DIGITAL HOME HEALTHCARE

Digital home healthcare can alleviate system burdens by replacing hospital visits and/or admissions, enabling continuous monitoring, early detection of complications, and personalized care tailored to the individual patient's needs. For patients, digital home healthcare offers convenience, reduces the need for hospital visits, and empowers them to manage their health. Providers benefit from real‐time data, enabling proactive interventions and efficient resource allocation.

Despite these promising outcomes, significant barriers hinder the large‐scale adoption of digital home healthcare:

1. Safety and Efficacy: Concerns persist about overdiagnosis, overtreatment, and the reliability of remote monitoring devices. Without robust scientific evidence, there is a risk of unnecessary medical interventions, undermining trust in digital solutions.
2. Funding and Reimbursement: Traditional healthcare financing systems are often ill‐suited to digital care models. Questions about who bears the costs of devices, data storage, and connectivity create uncertainty for providers and payers.
3. Integration into Care Pathways: Implementing digital healthcare requires systemic changes, from updating workflows to training staff and addressing patient concerns about privacy and data security. The lack of interoperability between digital platforms and electronic medical records further complicates this transition.
4. Equity and Accessibility: Socioeconomic disparities within and between regions or countries pose challenges to equitable adoption. Limited internet connectivity and digital illiteracy threaten to widen health disparities.

Premature adoption of digital home healthcare without robust evidence carries significant risks. Poorly designed systems may exacerbate health disparities, particularly for patients with limited digital access. Overdiagnosis and overtreatment could lead to unnecessary anxiety, medical interventions, additional strain for healthcare workers, and inflated costs. A cautious, evidence‐based approach is essential to ensure digital solutions deliver on their promise of safer, more efficient care.

## 2 THE ROLE OF RESEARCH

Research plays a pivotal role in overcoming these barriers. Well‐designed studies are needed to confirm the safety, efficacy, and scalability of digital health solutions across diverse populations. Assessing cost‐effectiveness is equally crucial to designing sustainable funding models. Value‐based reimbursement—rewarding outcomes rather than service volume—offers a promising framework for digital home healthcare. Furthermore, understanding both practical and psychological barriers and facilitators of systemic adoption is critical for scaling digital solutions. Research can identify best practices for integrating digital tools into existing workflows and addressing resistance among providers and patients.

## 3 REMOTE DIGITAL PREGNANCY CARE AS A USE CASE

There are about 4 million childbirths in Europe every year,^3^ and globally about 140 million.^4^ Several pregnancy complications may compromise maternal or fetal health and turn a pregnancy from low‐risk into high‐risk. Currently, around 10% of pregnancies across the EU are considered high‐risk, and this number is increasing.^5^ Hypertensive disorders of pregnancy (HDP) result in maternal complications but also affect fetal outcomes, including fetal growth restriction leading to low birth weight and preterm birth. These complications are associated with increased risk for maternal and perinatal mortality and with long‐term health implications and psychological effects for the mother, child, and their family.

To this day, many high‐risk pregnancies need hospitalization or a steep rise in outpatient clinic visits, usually extending through (pre‐term) delivery into the postpartum period. These care activities deeply impact patient experience and quality of life for the patient and their families, as well as increasing both healthcare costs and clinical workload. Even so, pregnant women seem to be the ideal population for remote digital care as they are relatively young, used to organizing their lives with their smartphone, and due to the work‐family life benefit, extra from restraining from hospital visits.

Several studies in pregnant women have shown the benefits from home monitoring of symptoms and blood pressure at home using remote blood pressure devices, supported by an online platform.^6^, ^7^, ^8^, ^9^ In the BUMP‐2 trial, 430 women with HDP in a self‐monitoring group were compared with 420 women receiving usual care.^6^ No significant differences in perinatal outcomes were found, confirming the safety of home monitoring. Several other studies confirmed feasibility and safety. The SAFE@home case–control study showed that blood pressure telemonitoring for women at risk of hypertensive complications allows fewer antenatal visits and hypertension‐related admissions, accompanied by a 19.7% net reduction in costs. Home monitoring was appreciated by patients with similar clinical outcomes.^8^, ^9^

The next step is home monitoring of the pregnant woman and her fetus in case of pregnancy complications often requiring hospital admission in preeclampsia, premature rupture of membranes, and fetal growth restriction. The fetal condition can be assessed using remote CTG devices tracing the fetal heart rate. In the HoTeL study, patients were randomized between home monitoring of fetal–maternal condition and hospital admission.^10^ During home monitoring, daily assessment of the remote CTG and maternal parameters was performed by clinical midwives. In case of alarm symptoms, the midwife referred the patient to the hospital. It was a requirement that the patient lived within a 30‐minute travel distance from a hospital. The study showed improved patient satisfaction, an 18% net cost reduction with equivalent clinical safety compared to traditional hospital care. In this study with a non‐inferiority design, the home‐monitoring group did not show inferior outcomes than the hospital group. While this randomized study showed promising results, more data are needed on the clinical safety of telemonitoring pregnancies. Moreover, knowledge is needed on how to overcome challenges impeding the implementation of remote digital pregnancy care for large‐scale uptake throughout the globe. In the meantime, tight safety arrangements are necessary for the use of digital technology.

## 4 A CALL TO ACTION

The time to act is now. Healthcare systems cannot afford to overlook the potential of digital home healthcare in addressing workforce shortages and rising costs. Governments and funding agencies must prioritize research into digital home healthcare, supporting clinical trials, economic evaluations, and implementation studies. Stakeholders should collaborate to create reimbursement frameworks that align incentives with outcomes and support the widespread adoption of digital health solutions. Also, technological support for the connection of remote devices and platforms to electronic medical records is crucial.

International collaboration in consortia may address challenges unique to different healthcare systems. By pooling expertise and resources, such initiatives can develop adaptable solutions for diverse healthcare settings. We have established the PregnaDigit‐EU consortium to enhance the potential of digital remote pregnancy care. PregnaDigit‐EU focuses on remote digital pregnancy care to monitor high‐risk pregnancies using advanced home‐monitoring devices, such as cardiotocography (CTG) for fetal health and blood pressure monitors for maternal health. By comparing clinical and implementation outcomes, patients' needs as well as reimbursement challenges between three European countries (the Netherlands, Sweden, and Spain) we aim to assess barriers and improve digital solutions for different populations.

## 5 CONCLUSION

Digital home healthcare holds immense promise in addressing the global challenges confronting healthcare systems. By enabling safe, effective, and patient‐centered care, it has the potential to transform care delivery. However, its successful implementation hinges on robust scientific evidence, sustainable funding models, and a deep understanding of diverse contexts.

The PregnaDigit‐EU project aims to offer a blueprint for the future of digital healthcare, demonstrating how research and innovation can overcome barriers to adoption. With concerted efforts and a strong commitment to collaboration, we hopefully help to unlock the full potential of digital home healthcare, paving the way for more accessible, sustainable, and equitable healthcare systems.
